# Can surveys of women accurately track indicators of maternal and newborn care? A validity and reliability study in Kenya

**DOI:** 10.7189/jogh.06.020502

**Published:** 2016-12

**Authors:** Katharine J McCarthy, Ann K Blanc, Charlotte E Warren, James Kimani, Brian Mdawida, Charity Ndwidga

**Affiliations:** 1 Population Council, New York, NY, USA; 2 Population Council, Washington D.C., USA; 3 Department for International Development, London, UK; 4 Population Council, Nairobi, Kenya

## Abstract

**Background:**

Tracking progress on maternal and newborn survival requires accurate information on the coverage of essential interventions. Despite widespread use, most indicators measuring maternal and newborn intervention coverage have not been validated. This study assessed the ability of women delivering in two Kenyan hospitals to recall critical elements of care received during the intrapartum and immediate postnatal period at two time points: hospital discharge and 13–15 months following delivery.

**Methods:**

Women’s reports of received care were compared against observations by trained third party observers. Indicators selected for validation were either currently in use or have the potential to be included in population–based surveys. We used a mixed–methods approach to validate women’s reporting ability. We calculated individual–reporting accuracy using the area under the receiver operating curve (AUC), population–level accuracy using the inflation factor (IF), and compared the accuracy of women’s reporting at baseline and follow–up. We also assessed the consistency of women’s reporting over time. We used in–depth interviews with a sub–set of women (n = 20) to assess their understanding of key survey terms.

**Results:**

Of 606 women who participated at baseline and agreed to follow–up, 515 were re–interviewed. Thirty–eight indicators had sufficient sample size for validation analysis; ten met criteria for high or moderate reporting accuracy (0.60<AUC) alone and ten met criteria for low population–level bias alone (0.75<IF<1.25). There was a significant decline in the individual level reporting accuracy between baseline and follow–up for ten indicators. Seven indicators had moderate or higher (0.4≤r_phi_) consistency between self–reports at baseline and follow–up. Four indicators met all criteria at follow–up: support person was present during the birth, episiotomy, caesarean section, and low birthweight infant (<2500 g).

**Conclusion:**

The few indicators that women reported accurately at baseline were consistently recalled with accuracy at 13–15 months follow–up. Although there is deterioration in women’s recall in some indicators over time, the extent of deterioration does not appreciably compromise reporting accuracy for indicators with high baseline validity. Indicators related to initial client assessment and the immediate postnatal period have generally low accuracy and poor reporting consistency over time.

Continued regional, national, and sub–national disparities in maternal and newborn deaths, 99% of which occur in low and middle–income countries (LMIC), underscore the need to accurately track progress in the coverage of proven lifesaving interventions [1]. Given that weak health systems infrastructures often characterize high mortality areas, measuring access to and the quality of maternal and newborn intrapartum and immediate postnatal care often relies on women’s responses to household survey questions, such as those included in the Multiple Indicator Cluster Surveys (MICS) and the Demographic and Health Surveys (DHS). Indicators of intrapartum care tracked in MICS and the DHS include facility–based delivery, skilled attendance at birth, the initiation of breastfeeding in the first hour of birth, and caesarean section. Such indicators are routinely used to track progress in maternal and newborn health. Nevertheless, the accuracy of intervention coverage data as measured through household surveys of female respondents has yet to be empirically established [2].

In response to the need for reliable data to guide maternal and newborn health efforts, several studies have sought to validate women’s reporting on indicators of the content of maternal and newborn health care in LMIC [3–6]. In general, these validation studies have found that a few concrete and particularly salient aspects of care, such as cesarean section [3,5,6], a support person present during the birth [3,6], a nurse–midwife provider during delivery [6], experience of hemorrhage [6], and low infant birthweight [6], can be accurately reported. The accuracy of reporting on other indicators, however, such as the initiation of breastfeeding, the practice of newborn skin–to–skin contact, and the administration of a uterotonic for the prevention of postnatal hemorrhage, has been shown to be high in some settings but not others, highlighting the need for further and context–specific research.

One limitation of extant validation research is that existing studies have not replicated completely the conditions of household survey programs such as the DHS and MICS that collect data on maternal and newborn intervention coverage. Women interviewed in these programs are asked to recall events related to a birth that took place within the two (or five) years prior to the survey; existing studies are not able to assess the extent to which women’s reporting accuracy and reliability may change as the time since the birth increases.

The present study addresses this gap in the evidence base by informing how women’s recall of maternal and immediate postnatal interventions changes over time. We conducted household interviews with women who had delivered in a Kenya hospital 13–15 months prior. To assess the validity and reliability of her recall, we compared women’s self–report at follow–up to: 1) observations by a third party at the time of labor and delivery, and 2) her previous exit interview at the time of hospital discharge. To elucidate factors that influence women’s reporting ability, in–depth interviews were conducted with a subset (N = 20) of respondents. Findings on the accuracy of women’s reporting at the time of hospital discharge has been previously published [6].

The main objectives of the study are: (1) to assess how accurately women report on the coverage of maternal and newborn health interventions received during the intrapartum and immediate postnatal period 13–15 months prior to the survey, (2) to examine the extent to which deterioration in recall compromises the validity of women’s reporting, and (3) to provide insight into factors that influence women’s ability to recall events surrounding the birth and understand survey questions.

## METHODS

### Participants

The sample population was comprised of women whose births were documented by research staff in study facilities at the time of delivery (July to September, 2013) and who provided consent and contact details to be visited for re–interview in their home approximately 13–15 months later (n = 609). Baseline data collection took place in two health facilities in Kisumu County and Kiambu County. Both study facilities are large public hospitals serving women who are either self–referred for care or who are referred from other health facilities due to high–risk pregnancies.

At baseline, women aged 15–49 years, who were admitted to the labor ward at the two study facilities were eligible for inclusion (n = 662; n = 388 in Kiambu, n = 274 in Kisumu) [6]. Participants were consecutively enrolled until the requisite sample size was reached. Women who provided written informed consent for participation completed an exit interview following delivery and prior to facility discharge. Further details on the study setting and methods for data collection for the reference standard are reported in our baseline paper [6].

At follow–up women who participated at baseline and who agreed to participate at follow–up were re–interviewed in their home or other mutually agreed upon location in the community (July to November, 2014).

### Test methods

Our reference standard for the study was direct observation by trained observers who used a structured checklist to document the care received and interactions between women and health providers in the maternal admission room and labor and delivery rooms. Study observers were registered nurse/midwives with at least three years of experience in maternal, newborn and child health. Direct observation reflected all aspects of caregiving and data collectors supplemented observations by asking providers or checking medical records if clarification was needed [6].

Interviews with women were administered by data collectors who were degree holders in social sciences. Study interviewers were women from the local area, fluent in the local dialects (Kiswahili, Dholuo and Kikuyu) and not the same individuals as the study observers. All data collectors received a four–day training on administering the interview protocol and the appropriate procedures for ethical research with human subjects.

### Questionnaire instruments

To assess changes in reporting accuracy over time, women were asked the same set of questions at follow–up as at the baseline hospital exit interview. Survey questions reflected key maternal and newborn interventions in the intrapartum and immediate postnatal period (upon admission to the labor ward until 1 hour following delivery). While the full process for indicator selection has been described previously [6], in brief, indicators to be assessed were identified by conducting a landscaping scan of published and grey literature in 2012. A total of 80 out of an initial list of 285 indicators were selected by a group of public health experts specializing in validity assessment. Where possible, question wording identical to that used in the DHS and MICS was used.

Several assessed indicators are included in global health initiatives such as the Global and National Targets 2020–2035 of the Every Newborn Action Plan and the WHO 100 Core Global Health Indicators [7,8]. Additionally, tracking of skilled birth attendance is proposed for inclusion in the Sustainable Development Goals [9], while the WHO Strategies towards Ending Preventable Maternal Mortality (EPMM) emphasizes documenting major causes that lead to maternal deaths [10]. Table S1 in **Online Supplementary Document(Online Supplementary Document)** indicates survey questions that correspond with elements of such global health initiatives. For example, given that the type of provider who is legislated to perform lifesaving functions varies by setting [11], it is essential to know how accurately women can identify the type of provider who assisted them. As such, this study assesses the accuracy of women’s reporting on the type of the main provider who assisted them during labor and delivery. Similarly, intravenous oxytocin is the standard of care recommended for the prevention of postpartum hemorrhage [12]. As a proxy to asking women the names of medications received during delivery (which women are less likely to be informed of), women were asked about all potential administration routes that medication might have been received within a few minutes following delivery.

A randomly selected subset of women who agreed to participate in the in–depth interview were asked open–ended questions related to their understanding of key terms and concepts included in the questionnaire. A sample size of 20 women was considered sufficient to gain insight into the most common factors that influence women’s understanding of the survey questions.

### Ethical review

Prior to re–interview, all women were provided with a description of the study, including the right to refuse participation with no consequence, or to stop the interview at any time. Participants were informed that they may be randomly selected to answer additional open–ended questions regarding their understanding of terms used in the interview questionnaire and that participation in both activities was voluntary. Only women who participated in the survey interview were asked to complete the in–depth interview. Written informed consent for both activities was obtained in the woman’s native language (Kiswahili, Dholuo, or Kikuyu) prior to participation. In Kenya, adolescents under the age of 18 who are pregnant or a parent are considered “emancipated minors” and are able to provide written informed consent [13–15].

The study and consenting procedures were approved by the Population Council [Protocol No. 594] and the Kenya Medical Research Institute (KEMRI) [Protocol No. 395], prior to participant enrollment.

### Analysis

Briefly, as described in the baseline study, a target sample of 600 women was sought. Sample size was calculated for a type one error level of 5%, sensitivity of 60 ± 6% precision, 70% specificity ± 6% precision, assuming 50% indicator prevalence and 20% attrition between baseline and follow–up [6,16].

Statistical analysis was performed using Stata Version 13 (StataCorp, College Station, TX, USA). We assessed validity and reliability of women’s responses at the individual–level as well as validity at the population–level. In order to assess changes in women’s reporting accuracy over time, all baseline analyses are restricted to women who participated at follow–up. To assess for the potential for systematic bias in the types of women lost to follow–up, we conducted chi–square tests and used the Holm–Bonferonni correction to adjust for multiple comparisons.

For individual–level validity, the sensitivity and specificity of women’s recall was computed for each indicator by first constructing two–by–two tables of women’s responses (Yes, No) vs the reference standard (Yes, No). “Don’t Know” responses were treated as “No” responses (ie, women were not positive that the intervention had occurred). Pairwise missing data were excluded from the analysis.

Next, we quantified the area under the receiver operating curve (AUC) for all indicators with sufficient sample size (at least 5 counts per cell) to summarize validity in a global statistic [3,6]. The AUC plots each indicator’s true positive rate (sensitivity) against its false positive rate (1 – specificity) to produce a summary estimate of validity [17]. Perfect indicator classification would have an AUC value of 1.0, while a random response would produce an AUC value of 0.5. AUC estimates were calculated to assess women’s reporting at follow–up compared to the observer classification (reference standard).

The change in the validity of women’s reporting over time was assessed by comparing follow–up and baseline AUC estimates using an equation which allows for tests of equality of two or more AUC estimates obtained from correlated samples [18,19]. For sensitivity, specificity and AUC values, corresponding 95% CI estimates are provided, assuming a normal approximation to the binomial distribution. We considered 0.70<AUC to reflect high accuracy; 0.60<AUC<0.70 as moderate accuracy, and AUC<0.60 as low accuracy [3,6].

We assessed indicator reliability at the individual–level by comparing women’s responses to survey questions administered at follow–up to their responses at baseline. The agreement between two binary responses is measured by the phi coefficient (r_phi_). The r_phi_ can range from –1 to 1, where 0 represents no correlation and 1 represents perfect agreement. The Dancey and Reid classification of correlation was used with r_phi_<0.40 indicative of poor agreement, 0.4≤r_phi_<0.6 moderate agreement, 0.6≤r_phi_<0.8 high agreement; 0.8≤r_phi_ for almost perfect agreement [20].

To assess population–level accuracy, we calculated the inflation factor (IF) (also known as the Test to Actual Positives ratio) [21]. The IF reflects the prevalence of the indicator that would be obtained by women in a survey (Pr) divided by its true prevalence (ie, observer report) (P). The prevalence based on women’s report in a survey (Pr) is calculated by applying each indicator’s estimated sensitivity (SE) and specificity (SP) to its true prevalence (P), using the following equation: Pr = P × (SE + SP – 1) + (1 – SP) [22]. The ratio of the indicator survey–based prevalence to its true prevalence estimates the extent each indicator would be over or under–estimated if obtained by survey self–report in the study setting (IF = Pr/P) [21,22]. We categorized the degree of bias reflected by the IF as low (0.75<IF<1.25), moderate (0.50<IF<1.5) and large (IF<0.50 or IF>1.5) [4,6]. Changes in population–level accuracy over time were assessed by comparing changes in IF classification between baseline and follow–up.

We summarize indicator performance in terms of both individual (AUC and r_phi_) and population–level (IF) reporting. Indicators which had moderate or higher individual–level accuracy and reliability, and low population bias are considered to have overall acceptable validity (0.60<AUC, 0.4≤r_phi_ and 0.75<IF<1.25). We caution readers that indicator usability depends on the purpose of measurement. An indicator with poor individual–level reporting may produce an acceptable estimate of population–level coverage if the ratio of false positive to false negative reports is approximately 1. We refer readers to the full–presented validation results.

For the analysis of qualitative interview data, individual in–depth interviews were audio recorded and transcribed verbatim. Transcripts were translated into English and imported into NVIVO 10 software for coding (QSR International Limited, London, UK). A codebook was developed a priori by the research team to assess main themes of interest. These themes related to women’s understanding of terms and concepts in survey questions that were reported with difficulty at baseline, including: how women ascertained the skill level of their provider, women’s understanding of ‘skin–to–skin’ practice for newborn thermal care, and understanding of the timing and sequencing of events, such as the term “immediately”. Two independent researchers familiar with the local context coded the transcripts. As a check for internal consistency, a subset of transcripts (n = 5) were compared and reconciled.

## RESULTS

### Sample description

**Figure 1:**
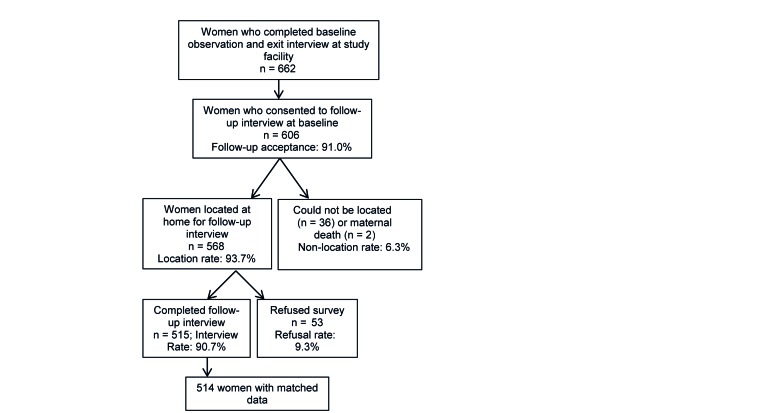
Participant enrollment.

Of the 662 women whose births were documented at baseline, 606 agreed to a follow–up interview (91%). Data collectors were able to locate 568 women and re–interview 515 women in their home community (85% follow–up of those who provided baseline consent) and complete matched data was obtained for 514 women (**Figure 1**). The majority of women re–interviewed resided in the two counties where the baseline hospital facilities were located.

**Table 1** presents the background characteristics of women who participated in the baseline and follow–up interviews, respectively. Participants who were lost to follow–up were more likely to have delivered in the Kisumu County facility than those who remained in the study (53% vs 38%) and were less likely to have delivered in the Kiambu County facility than those who remained in the study (47% vs 62%) (Pearson  chi–square: 11.4, *P* = 0.001). Women lost to follow–up were also less likely to have three or more prior births than those who remained (15% vs 26%) (Pearson  chi–square: 6.8, *P* = 0.009).

**Table 1:** Respondent background characteristics by attrition status

Characteristics	In baseline only (%, n = 150)	In baseline and follow–up (%, n = 514	*P*–value
**Facility:**			
Kisumu	53.3†	37.9†	0.001*
Kiambu	46.7†	62.1†	
**Age (in years):**			0.134
15–19	19.3	13.3	
20–24	40.0	41.0	
25–29	28.0	30.5	
30–34	8.7	8.6	
35–39	3.3	6.3	
40+	0.7	0.4	
**Parity:**			0.020*
1	59.3	48.2	
2	25.3	26.2	
3 or more	15.3^1^	25.6†	
**Education level:**			0.049*
None	13.3	9.4	
Primary	42.7	44.3	
Secondary	22.7	31.5	
Higher	21.3	14.8	
**Marital status:**			0.088
Single, never married	20.7	13.0	
Married / living together	78.0	85.0	
Separated/ divorced / widowed	1.3	2.2	
**Cesarean section**	14.0	13.3	0.820

*Based on chi–square test, statistically significant at *P* < 0.05.

†Statistically significant pairwise comparisons using the Holm–Bonferroni correction to adjust for multiple comparisons.

### Validation results

The percentage of women who responded to survey questions is an important reflection of recall ability. More than 5% of women responded “I Don’t Know” to 11 indicators at follow–up, compared to four indicators at baseline (**Table 2**). In general, there was a high degree of overlap between indicators with high levels of ‘Don’t Know’ responses at both time points. Women were least likely to recall events related to provider hygiene and the immediate postnatal period.

**Table 2:** Indicators with “Don’t Know” responses >5%

Survey question	Follow–up “Don’t Know” %	Baseline* “Don’t Know” %
Did the health provider(s) wash his/her hands with soap and water or use antiseptic before delivering your baby?	43.3 (n = 437)	36.2 (n = 445)
Did the health provider(s) wash his/her hands with soap and water or use antiseptic before examining you?	41.2 (n = 515)	32.4 (n = 512)
Was your baby dried off with a towel or cloth immediately after his/her birth?	13.3 (n = 513)	8.4 (n = 511)
Why did you decide to delivery in this facility?	8.7 (n = 515)	0.0 (n = 512)
After the delivery of your baby, in the first few minutes after the delivery of the placenta, did anyone give you an injection in your thigh?	8.5 (n = 437)	4.5 (n = 445)
Were you allowed to drink liquids or eat any foods while you were in labor?	7.2 (n = 515)	4.7 (n = 512)
Just after the delivery of your baby, in the first few minutes after the delivery of your baby, did anyone give you an injection in your thigh or buttock?	6.2 (n = 437)	1.1 (n = 445)
In the first physical examination/ check after delivery, did a health provider do a perineal exam?	5.8 (n = 514)	10.6 (n = 512)
Did you or anyone else give anything to the baby other than breastmilk to eat or drink within the first hour after delivery?	5.7 (n = 513)	3.5 (n = 512)
Did someone place the baby on your chest, against your skin, immediately after delivery of the baby?	5.3 (n = 513)	2.9 (n = 512)

*Women who participated in both baseline and follow-up only.

**Reporting accuracy.** Of the 57 indicators measured, 38 had sufficient variation for robust analysis (**Table 3**). Of these, four indicators met our criteria for both moderate or higher individual validity, reliability and low population–level bias at follow–up: (1) a support person was present during the birth, (2) episiotomy, (3) cesarean section, and (4) low birthweight infant (**Table 3**). All but the episiotomy indicator also met the AUC and IF criteria at baseline.

**Table 3:** Summary of validation and reliability results*

Indicator	Individual–level accuracy (0.60<AUC: ✓)	Population–level accuracy (0.75<IF<1.25: ✓)	Test–retest reliability (0.4≤r_phi_: ✓)
**Baseline | Follow–up**	**Baseline | Follow–up**
**Initial client assessment:**			
Takes blood pressure	– | –	✓ | ✓	–
HIV status checked	– | NA†	– | –	–
Receives HIV test	– | NA	– | ✓	–
Provider washes hands with soap and water or uses antiseptic before any initial examination	– | –	– | –	–
**Provider respectful care:**			
Encourages/assists woman to ambulate during labor	– | –	✓ | ✓	–
Allowed to drink liquids/eat	– | –	✓ | –	–
Encourages/assists woman to assume different positions in labor	– | –	– | –	–
Allowed to have a support person present	– | –	– | –	–
A support person is present during birth	✓ | ✓	✓ | ✓	✓
**First stage of labor:**			
Induces labor with a uterotonic (IV line, injection or tablet)	✓ | ✓	– | –	–
Augments labor with a uterotonic (IV line, injection or tablet)	✓ | ✓	– | –	✓
Uterotonic received some time before birth (to induce or augment labor)	✓ | ✓	– | –	✓
Performs artificial rupture of the membranes	– | –	– | –	–
**Skilled birth attendance:**			
Main provider delivery– skilled (doctor, medical resident or nurse/midwife)	– | –	✓ | ✓	–
Main provider delivery–doctor or medical resident	✓| ✓	– | –	–
Main provider delivery–nurse/midwife	✓| ✓	✓ | ✓	–
**Second & third stage labor:**			
Uterotonic administered in 1–3 min following delivery (injection, IV line or tablets) (women who had vaginal delivery)	– | NA	✓ | NA	–
Uterotonic administered after delivery of placenta (women who had vaginal delivery)	– | –	– | –	–
Method of uterotonic post–birth by injection (women who had vaginal delivery)	✓ | NA	✓ | NA	–
Episiotomy	✓ | ✓	– | ✓	✓
Cesarean section	✓ | ✓	✓ | ✓	✓
**Immediate postnatal care–newborn:**			
Newborn placed with mother immediately following birth (all women)	– | –	✓ | ✓	–
Breastfed infant in first hour after birth	– | ✓	✓ | ✓	–
Skin to skin	– | –	– | –	✓
3 essential elements of newborn care (immed. dried + newborn placed immediately skin to skin with mother + breastfed within 1 hour of birth)	– | –	– | –	–
**Immediate postnatal care–mother:**			
Uterine massage after delivery of placenta (denominator: vaginal delivery)	– | –	✓ | ✓	–
In first examination post–delivery, did provider ask or check for bleeding?	– | –	✓ | ✓	–
In first examination post–delivery, did provider examine perineum?	– | –	– | ✓	–
In first examination post–delivery, did provider take temperature?	– | ✓	✓ | ✓	–
In first examination post–delivery, did provider take blood pressure?	– | ✓	✓ | –	–
In first examination post–delivery, did provider check for involution?	– | –	✓ | ✓	–
**Maternal and newborn morbidity:**			
Low birthweight infant (<2500 g)	✓ | ✓	✓ | ✓	✓
Complications – hemorrhage	✓ | ✓	– | –	–
Complications – prolonged labor	✓ | ✓	– | –	–
Complications – none	✓ | –	✓ | ✓	–
Complications – yes (to any)	✓ | –	– | –	–
Asked for pain relief medication	– | –	– | –	–
Received pain relief medication	✓ | ✓	– | –	✓

*Note: See Table S1 in **Online Supplementary Document(Online Supplementary Document)** for full description of survey questions that comprise each indicator.

†NA results had insufficient data (n<5 in cell of two by two table) for robust analysis in respective survey round; indicators with insufficient sample size at both baseline and follow-up are omitted from table. Horizontal dash (–) indicates that the acceptability threshold was not met.

In total, seven of 38 indicators are classified as having high validity, eight as moderate and 23 as low. There was a statistically significant deterioration in reporting accuracy for 10 indicators between baseline and follow–up (Table S2 in **Online Supplementary Document(Online Supplementary Document)**). There was an additional significant increase in AUC for one indicator (membrane rupture) that is likely attributable to random fluctuation due to its low validity at both time points. Five indicators with high validity at baseline significantly declined at follow–up; however, only two declined enough to lose the high validity classification at follow–up. These were: (1) injection received at some time before the birth (proxy for uterotonic to induce or augment labor), and (2) injection received to strengthen labor (proxy for uterotonic to augment labor). These indicators fell to moderate validity at follow–up.

Three indicators that had a moderate baseline AUC level significantly declined to low validity AUC level at follow–up: (1) allowed to have a support companion present, (2) blood pressure taken at first postnatal physical exam, and (3) temperature taken at first postnatal physical examination. There were significant differences between baseline and follow–up AUC levels for three indicators with low baseline reporting accuracy; all of these indicators retained low validity at follow–up. These were: (1) artificial rupture of the membranes performed, (2) provider encourages or assists woman to ambulate during labor, and (3) the provider checked for bleeding in the first postnatal physical examination.

**Reliability.** Across all indicators, women’s reporting consistency was generally poor (31 of 38 indicators had r_phi_<0.40). Only seven indicators met the criteria for moderate to high reliability (Table S3 in **Online Supplementary Document(Online Supplementary Document)**). The consistency of reporting of caesarean sections was nearly perfect (r_phi_ = 0.90), while low birthweight infant and episiotomy had substantial agreement (0.6≤r_phi_<0.8). There was moderate agreement (0.4≤r_phi_<0.6) for four indicators: (1) skin–to–skin contact of mother and newborn following birth, (2) injection received some time before birth (ie, proxy for uterotonic to induce or augment labor), (3) a support person present during birth, and (4) injection received to strengthen labor (ie, uterotonic to augment labor). Results show poor reliability for indicators related to the type of provider, immediate postnatal care for the mother, and complications.

**Population–level bias.** Table S2 in **Online Supplementary Document(Online Supplementary Document)** presents the prevalence of intervention coverage that would be obtained from women’s reports in a household survey given the specificity and sensitivity observed in the follow–up survey. In total, 14 indicators met the criteria for low bias in population–level coverage estimates at follow–up, three indicators had moderate population–level bias and 21 indicators had large bias.

The top five indicators with the largest predicted overestimation of self–reported prevalence from a household survey of women (IF>1.5) at follow–up related to the immediate postnatal period, including receiving an injection following the delivery of the placenta (proxy for uterotonic for the preventing of postnatal hemorrhage), immediate newborn care, and complications. Indicators with the largest predicted underestimation related to some aspects of care in which the provider may not have been explained the purpose to women, such as “*In the first physical examination after delivery, did a health provider do a perineal exam?”* and *“Did a health provider check your belly to see if your womb was becoming firm after the birth of your baby*?” and “HIV status checked”, which may have been done by checking records rather than by asking women.

For the majority of indicators (29 of 38) IF levels at follow–up did not change appreciably from baseline. Specifically, five of the 12 indicators with low bias at baseline, five of the nine with moderate bias and 17 of the 18 with large bias remained in the same classification category at follow–up. Of the eight indicators that changed classification categories: four indicators changed to a higher bias category and four indicators changed to a lower bias category at follow–up. Ten indicators had large magnitude changes, an IF difference of greater than 0.5. All large magnitude changes occurred among high baseline IF indicators.

**Qualitative results.** Qualitative data provide insight into what women recall about the labor and delivery process at 13–15 months postnatal, as well as their understanding of terms and concepts used in survey questions. When describing their hospital delivery experience during the in–depth interviews, women often mentioned emotions and physical experiences. These included fears about having a healthy delivery and the pain of labor.

“*I just wanted to give birth normally and successfully*.”

“*The labor was so intense you cannot even tell the aspects of care you received… I was feeling bad, when in labor pains you just feel bad…The only thing on my mind is what I would give birth and rest*.”

Many respondents also reported experiencing fatigue, relief and joy following delivery. In some instances, these experiences may have outweighed recall of an intervention received during this period.

“*I felt so blessed to be alive though I had not seen my baby. When I was given the baby I didn’t have the strength to look at the baby because I was still in much pain*.”

“*You know that time* [after the birth] *I was over excited so after the caesarean section I was happy to see my child like this and I gave God my thanks, so I cannot know because once I saw the baby I was tired so whatever happened after that I don’t know*”.

“…*When you asked me if the baby was placed on my chest against my skin, that was hard for me to remember because at that time I was tired because I had gone without sleep for two days.*”

“*Like when you asked if I was injected after delivery, yes I can remember I was injected only once, but not sure if it was after delivery of baby or placenta… And you know* [I] *am always so afraid of injections but the joy* [of giving birth] *made me forget about the pain and the fear of injection*.”

Despite the influence of a woman’s physical state on her recall, interventions considered of immediate importance to her health, either by facilitating a healthy delivery (eg, inducing/augmenting labor or having a cesarean operation) or treating a complication, remained memorable. One aspect of care viewed as critical to ensuring a healthy delivery was who assisted with the delivery. For example, when asked to recall a particularly memorable aspect of care, one respondent reported, “*The doctor who assisted me to deliver because she gave me glucose to get more energy to push the baby*”. Another woman noted, “*The one who performed the surgery is the one who helped me the most*.”

In some instances support companions were also found to deliver needed care. As one respondent reported, *“If your relatives have not yet arrived to visit you suffer a lot because they do not give anything to eat or drink.”*

Aspects of care deemed by women to be less critical to their health were less readily recalled.

“*Issues of the nurses washing their hands, I could not remember what I remember is just them wearing the gloves…. Because I was not keen to check if they were doing that, to me gloves are just enough.*”

A defining characteristic used by women to differentiate higher vs lower skill level providers was the ability of the provider to ensure that she received the needed elements of care. As illustrated below, there was generally agreement on the interventions higher cadre providers were able to administer relative to lower level providers (ie, prescribe drugs, perform surgeries, and manage complications).

“*During my delivery a doctor came and said I should be given tablets… I was given five of them. He said that the tablet is not working on her so they were told to put IV on me. To me I thought that is the senior doctor*.”

Distinctions between qualified providers and medical students were more apparent as women reported relying on a variety of clues such as uniform, seniority and the types of services provided. However, many participants noted difficulty in discerning between types of providers considered ‘qualified’, such as doctors or nurses, if both were able to provide the needed care.

“*I knew it was a doctor because she is the one who tested me ruptured my membrane to assist the baby to come out, took my blood pressure and then tested my urine… I knew this is a doctor it is not a TBA or a student. [Interviewer: Okay, how do you differentiate a doctor and a nurse?] A doctor and a nurse, that one is hard*.”

“*That is the only one I can say the rest ask me the difference of the student but the rest is it is hard to tell who is a doctor and a nurse.*”

“Sometimes he makes noise to the other telling them that is not what should be done. So I knew that is a doctor but I am not able to differentiate a doctor and a nurse.”

Women’s understanding of the terms used in survey questions also influenced reporting. The concept of timing, for example, was invoked in several questions. Timing is critical to measuring access to lifesaving interventions such as postnatal interventions for the newborn, such as newborn thermal care. For example, women were asked, “*Did someone place the baby on your chest, against your skin, immediately after delivery of the baby?”* When asked to define the number of minutes that would have passed in an ‘immediate’ time frame, responses varied widely. One participant states, “*… let us say immediately because it didn’t pass twenty minutes or so*”. Others report, “…*immediate means just now, just a few minutes, one or two minutes*” and “*when you talk about immediately it should be five to seven*.” These results suggest indicators in which accurate reporting on timing is a necessary element may not be able to be reported with accuracy.

## DISCUSSION

Findings from this study show that the relatively few indicators that women are able to report with high accuracy at baseline are recalled with moderate or high accuracy after 13–15 months. Although the findings demonstrate that recall accuracy for some indicators declines with time, high validity indicators are more likely to be reliably reported by women and retain moderate–to–high accuracy at follow–up. Specifically, 6 of the 7 moderate–to–high reliability indicators (0.4≤r_phi_) also had moderate–to–high diagnostic accuracy (0.60<AUC). These results suggest that the more salient the intervention or event, the more accurately and reliably the indicator can be recalled over time.

Indicators with overall validity (0.60<AUC and 0.75<IF<1.25) were mostly related to aspects of care received between the first stage of labor and the birth. It is notable that no indicators related to the initial client assessment phase or immediate postnatal care for the mother or newborn met our criteria for moderate or high diagnostic accuracy, and all but one indicator (skin–to–skin contact of the mother and newborn) had low reliability. Furthermore, nearly all indicators with greater than 5% “Don’t Know” responses were related to these two phases. For example, the indicator of provider hand–washing or antiseptic use, had a >30% “Don’t Know” response and is not recommended in the current setting. The highest validity indicators related to particularly memorable aspects of birth either due to pain (eg, caesarean section, episiotomy), because they were considered significant to having a health delivery (eg, an intervention received in order to bring on (induce) or strengthen (augment) labor, a nurse/midwife or doctor/medical resident was the main provider present), or brought emotional support and comfort (eg, a support person was present).

For most indicators, however, we found women’s reports had low diagnostic accuracy (22 of 38 indicators have AUC<0.60), large population–level bias (23 indicators have IF<0.5 or 1.5<IF), and poor reliability (31 of 38 indicators have r_phi_<0.40). The low validity of indicators not immediately pertinent to the event of birth or health status of the mother or newborn may be due to the high background ‘noise’ of experiences preceding the first stage of labor and immediately following birth. As suggested from the qualitative findings, women’s recall of practices in the initial client assessment period may be clouded by the anxiety of labor and delivery, while the fatigue, pain, and joy associated with birth may interfere with recall of the care received immediately postnatal. For example, questions about receiving an injection *before* labor (to induce or augment labor) were recalled with moderate or high accuracy, while questions related to an injection immediately *following* birth (ie, uterotonic for prevention of postnatal hemorrhage) had mixed results and high “Don’t Know” responses. Taken together, we advise caution when assessing the coverage of interventions received immediately postnatal, especially when recall of timing is key. When self–reported data are used, we recommend the use of multiple questions regarding when and how the intervention was received in order to triangulate findings to enhance internal validity.

Despite generally poor reporting consistency by women, particularly in the early and late phases of birth, reporting discrepancies did not result in statistically significant changes in AUC levels or IF classification from those at exit interview for the majority of indicators. Where significant changes in individual level accuracy did occur (11 of 38 indicators), women were less accurate (10 of 11 indicators) over time. In contrast, we found that where changes in population–level bias occurred, IF estimates were equally likely to become larger or smaller with time. The discrepancy in the direction of change for these measures is due to the fact that while the AUC reflects individual–level accuracy, the IF reflects the balance of true positives and false negatives at the aggregate level. An indicator that meets criteria for the IF but not the AUC, such as ‘skilled birth attendance’ may generate an acceptable estimate of intervention coverage at the population level. Results from this study should be interpreted with respect to whether the goal is individual or population level measurement.

Findings from this study confirm the findings of prior literature; many indicators of intrapartum care and associated morbidities have generally low validity and reliability when assessed by women’s reports [3,23,24] but some salient indicators are reported with accuracy. Of the four indicators that met both validation criteria in this study, a support person present during birth [3], and cesarean section [4,5] have also been found to be reported accurately by women in prior studies.

The low reliability of women’s reports of complications corresponds with the conclusion of a 2012 review of several validation studies conducted in LMIC which found that the reliability of self–reported complications based on women’s recall is poor, even if the woman suffered from a life–threatening complication [25]. A study of similar design in Benin that compared clinic exit interviews (within 1 week of discharge) and interview responses of women at six months postnatal also found self–reported data to be neither reliable nor valid for measuring obstetric complications [24]. That all complication related indicators in the present study had large IF bias may be in part due to their low observed prevalence. A limitation of the IF is that when the coverage of a given quality of care indicator is low, even a small false positive rate will result in a biased IF estimate. Therefore, estimates of population–level survey results from this study suggest that self–reported data on rare labor and delivery events, such as the prevalence of complications, will be overestimated, as documented in prior studies [26]. To assess population–level bias in other contexts where intervention coverage may vary, one can model the estimated survey prevalence by applying the sensitivity and specificity calculated in this study to the estimated ‘true’ prevalence of the intervention for the given context, as detailed in the Analysis section. We refer readers to our previous article, which illustrates the implications of IF estimates for other contexts [6].

This study provides insight into the potential of self–reported data to assess accurately maternal and newborn health intervention coverage. The strengths of the study are the use of direct observation as the reference standard and the longitudinal study design, in which the re–interview of women 13–15 months postnatal more closely reflects conditions of household survey programs, such as the DHS and MICS.

We also note several limitations. For example, women participating in standard household surveys are typically not interviewed twice, and the recall of participants may have been influenced by repeated measurement. Furthermore, the 13–15–month recall period does not reflect the range of plausible recall periods of the MICS and DHS, which ask women to report on a birth that occurred within the preceding two or five years. Results from this study are reflective of women who delivered in the two study facilities, and may not be generalizable to other contexts. The majority of births take place in a facility and are delivered by a skilled provider in both study counties [27]. However, women who reside in rural areas, have lower education and less wealth are less likely to deliver in a facility and our results are less likely to reflect the reporting patterns of this population. As noted in the baseline study [6], the fact that the standard of care for both facilities was consistently high, also limited the ability to validate all indicators due to lack of variation in received care. Additional validation research that takes place across facility settings and time points is warranted.

Despite the limitation of this study in terms of facility setting, 61% of births in Kenya took place in a health facility in 2014 [27]. With the announcement of the Kenya Government to provide free maternity services in all public facilities in 2013 [28], this percentage is likely to continue to increase in the coming years and may extend the utility of the study results.

## CONCLUSION

Women are able to report on some aspects of maternal and newborn intervention coverage with accuracy. Results from this study do not suggest that there is significant deterioration in women’s recall ability over time for indicators that are reported with accuracy at baseline. Results confirm that the population–level coverage of low prevalence indicators is challenging to measure accurately. We found generally poor accuracy and reliability for indicators related to interventions received during the initial client assessment and immediate postnatal care for both the mother and newborn, which may result from high background ‘noise’ of physical and emotional experiences relative to the intervention. If self–reported data are used to measure intervention coverage in these periods, particularly if time is an essential element of the received care, we recommend caution and triangulation with other data sources.
