# Supplementary material for: Can surveys of women accurately track indicators of maternal and newborn care? A validity and reliability study in Kenya
Source: J Glob Health. 2016 Sep 3;6(2):020502. doi: 10.7189/jogh.06.020502 (PMC5012235; doi:10.7189/jogh.06.020502)
Supplement: Online Supplementary Document [file jogh-06-020502-s001.pdf]

# Online Supplementary Document

**McCarthy et al. Can surveys of women accurately track indicators of maternal and newborn care? A validity and reliability study in Kenya**

**J Glob Health 2016;6:020502**

**Table S1.** Survey indicators and questions.

| Indicator | Interview question | Inclusion in Global Health Initiative[a] |
|---|---|---|
| **Initial Client Assessment** | | |
| Takes blood pressure– initial examination | Did someone take your blood pressure, at or near your admission to the facility? | |
| HIV status checked | During your time at the health facility for the birth of your baby, did someone ask you what your HIV status was? | |
| Receives HIV test | While you were at the health facility for the birth of your baby, did anyone test you for HIV? | |
| Providers washes hands with soap and water or uses antiseptic before any initial examination | Did the health provider(s) wash his/her hands with soap and water or use antiseptic before examining you? | |
| **Provider Respectful Care** | | |
| Encourages/assists woman to ambulate during labor | Were you allowed to get up and walk around while you were in labor? | |
| Allowed to drink liquids/ eat | Were you allowed to drink liquids or eat any food while you were in labor? | |
| Encourages/assists woman to assume different positions in labor | Did a health care provider ask you what position you preferred during labor? | |
| Allowed to have a support | Were you allowed to have a companion with you | ENAP: Birth companion of choice and skilled |

| Indicator | Interview question | Inclusion in Global Health Initiative[a] |
|---|---|---|
| person present during labor and delivery | during your labor? [Or] Were you allowed to have a companion with you during your delivery? | attendant at birth. (Number of births attended by a doctor/ nurse/ midwife and where the mother had a birth companion of choice) |
| A support person/companion is present during birth | Did you have a companion with you during labor? [Or] Did you have a companion with you during delivery? | |
| **First Stage of Labor** | | |
| Induces labor with a uterotonic (IV, IM, tablet) | (1) For the birth of your baby, did your labor start spontaneously or did someone do something to bring on your labor? [Yes, No] (2) For your birth of your baby, what was done to bring on your labor? [Injection in thigh/buttocks, IV line inserted in arm, Given tablets to put under tongue, tablets inserted in birth canal, or tablets inserted in rectum] | |
| Augments labor with a uterotonic (IV, IM, tablet) | (1) For your delivery, was anything done to speed up or to strengthen your labor? (Yes, No) (2) What was done to speed up or to strengthen your labor? [Injection in thigh/buttocks, IV line inserted in arm, Given tablets to put under tongue, Tablets inserted in birth canal, or Tablets inserted in rectum] | |
| Uterotonic received some time before birth (to induce or augment labor) | Yes to induction OR yes to augmentation of labor indicator. | |
| Performs artificial rupture of the membranes | (1) For your delivery, was anything done to speed up or to strengthen your labor? (Yes, No) (2) What was done to speed up or to strengthen your | |

| Indicator | Interview question | Inclusion in Global Health Initiative[a] |
|---|---|---|
| | labor? [Membranes ruptured] | |
| **Skilled Birth Attendance** | | |
| Main provider delivery– skilled | Who was the main provider assisting you during delivery? [Doctor (ob–gyn), Medical resident or Nurse/midwife][b] | SDGs: Proportion of births attended by skilled health personnel<br>WHO100: Births attended by skilled health personnel<br>ENAP: (Number of births attended by a doctor/ nurse/ midwife)<br>DHS & MICS Survey Question: Who assisted with the delivery of (NAME)? |
| Main provider delivery– doctor or medical resident | Who was the main provider assisting you during delivery? | |
| Main provider delivery– nurse/midwife | Who was the main provider assisting you during delivery? | |
| | **Second & Third Stage Labor** | |
| Uterotonic administered in 1–3 minutes following delivery (injection, IV medication, tablets) (women who had vaginal delivery) | *Just after the delivery of your baby* In the first few minutes after the delivery of your baby, did anyone give you an injection in your thigh or buttock?<br>[Or] … did anyone give you medication intravenously (through a tube in your arm)?<br>[Or] ...did anyone give you tablets to swallow or hold in your mouth?<br>[Or] ...did anyone place tablets in your rectum? | |
| Uterotonic administered after delivery of placenta (women who had vaginal delivery) | After the delivery of your baby, in the first few minutes *after the delivery of the placenta*, did anyone give you an injection in your thigh?<br>[Or] ...did anyone give you tablets to take or hold in your mouth? | |
| Method of uterotonic post–birth– IM injection (women who had vaginal delivery) | After the delivery of your baby, in the first few minutes after the delivery of the placenta, did anyone give you an injection in your thigh? | |

| Indicator | Interview question | Inclusion in Global Health Initiative[a] |
|---|---|---|
| Episiotomy | For your delivery, shortly before you delivered your baby, did anyone cut the opening of your vagina (episiotomy) to make more room for the baby's head? | |
| Cesarean section | Was your baby delivered by cesarean, that is, did they cut your belly open to take the baby out? | DHS & MICS Survey Question: Was (NAME) delivered by caesarean, that is, did they cut your belly open to take the baby out? |
| **Immediate Postnatal Care– Newborn** | | |
| Newborn placed with mother immediately following birth (all women) | Was your baby given to you immediately, within a few minutes, after his/her birth? | ENAP: Proportion of newborns who received all four elements of essential newborn care: immediate and thorough drying, immediate skin–to–skin contact, delayed cord clamping, initiation of breastfeeding in the first hour. |
| Breastfed infant in first hour after birth | Did you breastfeed your baby within the first hour after delivery? | WHO100: Early initiation of breastfeeding (percentage of infants breastfed within 1 hour of birth)<br>ENAP: Early initiation of breastfeeding. (Number of babies fed within 1 hour of birth)<br>DHS & MICS Survey Question: How long after birth did you first put (NAME) to the breast? |
| Skin to skin newborn care | Did someone place the baby on your chest, against your skin, immediately after delivery of the baby? | ENAP: Proportion of newborns who received all four elements of essential newborn care: immediate and thorough drying, immediate skin–to– |

| Indicator | Interview question | Inclusion in Global Health Initiative[a] |
|---|---|---|
| | | skin contact, delayed cord clamping, initiation of breastfeeding in the first hour. |
| 3 essential elements of newborn care (immediately dried, skin to skin, breastfed) | (1) Was your baby dried off with a towel or cloth immediately after his/her birth, within a few minutes of delivery? [AND]<br>(2) Did someone place the baby on your chest, against your skin, immediately after delivery of the baby? [AND]<br>3) Did you breastfeed your baby within the first hour after delivery? | ENAP: Proportion of newborns who received all four elements of essential newborn care: immediate and thorough drying, immediate skin–to–skin contact, delayed cord clamping, initiation of breastfeeding in the first hour. |
| | **Immediate Postnatal Care– Mother** | |
| Uterine massage after delivery of placenta (women who had vaginal delivery) | After the delivery of the placenta, did the birth attendant firmly massage your lower abdomen to help make your womb contract (become firm)? | |
| In first examination post–delivery, did provider ask or check for bleeding? | In your first physical examination/check after delivery, did a health provider look for or ask you about bleeding? | |
| In first examination post–delivery, did provider examine perineum? | In your first physical examination/check after delivery, did a health provider do a perineal exam? | |
| In first examination post–delivery, did provider take temperature? | In your first physical examination/check after delivery, did someone take your temperature? | |
| In first examination post–delivery, did provider take blood pressure? | In your first physical examination/check after delivery, did someone take your blood pressure? | |

| Indicator | Interview question | Inclusion in Global Health Initiative[a] |
|---|---|---|
| In first examination post–delivery, did provider check for involution? | In your first physical examination/check after delivery, did a health provider check your belly to see if your womb was becoming firm after the birth of your baby? | |
| **Maternal and Newborn Morbidity** | | |
| Low birthweight infant (<2500g) | What was your baby's weight? [weight given in grams] | WHO100: Incidence of low birthweight among newborns (less than 2500 g)<br>DHS & MICS Survey Question: How much did (NAME) weigh? |
| Complications– hemorrhage | Did you experience any of the following complications during or within the first few hours after your delivery? [Excessive bleeding] | EPMM: Address all causes of maternal mortality, reproductive and maternal morbidities and related disabilities. Includes emphasis on documenting major causes and complications that lead to maternal death in every country. |
| Complications– prolonged labor | Did you experience any of the following complications during or within the first few hours after your delivery? [Long labor, more than 12 hours] | |
| Complications– none | Did you experience any of the following complications during or within the first few hours after your delivery? [None] | |
| Complications– yes (to any) | Did you experience any of the following complications during or within the first few hours after your delivery? [Yes to any] | |
| Asked for pain relief medication | Did you ask for pain relief at any point during your time at the health facility? | |
| Received pain relief medication | Did anyone give you any pain relief at any point during your time at the health facility? | |

[a] Related global health initiatives are Sustainable Development Goals (SDGs), Ending Preventable Maternal Mortality (EPMM), Every Newborn Action Plan (ENAP), and the WHO 100 Core Health Indicators (WHO 100) or included in population–based household survey programs the Demographic and Health Surveys (DHS) or Multiple Indicator Cluster Survey (MICS).
[b] 'Skilled birth attendance' indicator was constructed in analysis to combine women's responses of a doctor, nurse/midwife or medical resident as compared to all other response categories.

**Table S2.** Full validation results.[a]

| Indicator | Matched N at Baseline | Sensitivity (95% CI) of Follow–up Responses | Specificity (95% CI) of Follow–up Responses | True Prev (%) Observed at Baseline | Population–Based Survey Estimate at Follow–up | Matched N at Follow–up[b] | AUC at Follow–up (95% CI) | AUC at Baseline (95% CI) | IF at Follow–up | IF at Baseline | Met AUC and IF at Follow–up? [c] |
|---|---|---|---|---|---|---|---|---|---|---|---|
| **Initial Client Assessment** | | | | | | | | | | | |
| Takes blood pressure | 509 | 79.7 (75.8–83.3) | 17.1 (7.2–32.1) | 91.9 | 80.0 | 506 | 0.48 (0.44–0.53) | 0.53 (0.49–0.58) | 0.87 | 0.94 | No |
| HIV status checked | 514 | 40.8 (36.3–45.3) | 72.7 (54.5–86.7) | 93.6 | 39.9 | 511 | 0.57 (0.52–0.61) | NA | 0.43 | 0.25 | No |
| Receives HIV test | 513 | 13.2 (5.5–25.3) | 81.5 (77.7–85.0) | 10.3 | 17.9 | 506 | 0.47 (0.43–0.52) | NA | 1.74 | 0.81 | No |
| Provider washes hands with soap and water or uses antiseptic before any initial examination | 514 | 43.9 (34.6–53.5) | 59.8 (54.8–64.6) | 22.2 | 41.1 | 511 | 0.52 (0.47–0.56) | 0.58 (0.53–0.62) | 1.85 | 2.13 | No |
| **Provider Respectful Care** | | | | | | | | | | | |

**Table S2.** Full validation results.[a]

| Indicator | Matched N at Baseline | Sensitivity (95% CI) of Follow–up Responses | Specificity (95% CI) of Follow–up Responses | True Prev (%) Observed at Baseline | Population–Based Survey Estimate at Follow–up | Matched N at Follow–up[b] | AUC at Follow–up (95% CI) | AUC at Baseline (95% CI) | IF at Follow–up | IF at Baseline | Met AUC and IF at Follow–up? [c] |
|---|---|---|---|---|---|---|---|---|---|---|---|
| **Initial Client Assessment** | | | | | | | | | | | |
| Encourages/assists woman to ambulate during labor | 505 | 80.3 (76.0–84.1) | 19.1 (12.4–27.5) | 77.2 | 80.4 | 502 | 0.50* (0.45–0.54) | 0.58 (0.53–0.62) | 1.04 | 1.09 | No |
| Allowed to drink liquids/ eat | 509 | 66.5 (59.6–72.9) | 48.2 (42.4–54.0) | 40.5 | 57.8 | 506 | 0.57 (0.53–0.62) | 0.56 (0.52–0.61) | 1.43 | 1.50 | No |
| Encourages/assists woman to assume different positions in labor | 509 | 11.2 (7.8–15.4) | 92.4 (88.1–95.5) | 56.2 | 9.6 | 506 | 0.52 (0.47–0.56) | 0.54 (0.49–0.58) | 0.17 | 0.22 | No |
| Allowed to have a support person present | 508 | 31.1 (18.2–46.6) | 76.7 (72.6–80.5) | 8.9 | 24.0 | 504 | 0.54* (0.49–0.58) | 0.64 (0.59–0.68) | 2.71 | 2.20 | No |
| A support person is present during birth | 507 | 34.6 (17.2–55.7) | 97.3 (95.4–98.6) | 5.1 | 4.3 | 504 | 0.66 (0.62–0.70) | 0.76 (0.72–0.80) | 0.85 | 0.88 | Yes |
| **First Stage of Labor** | | | | | | | | | | | |
| Induces labor with a uterotonic (IV, IM, | 499 | 63.6 (40.7–82.8) | 85.3 (81.8–88.4) | 4.4 | 16.8 | 496 | 0.74 (0.70–0.78) | 0.84 (0.80–0.87) | 3.82 | 2.71 | No |

**Table S2.** Full validation results.[a]

| Indicator | Matched N at Baseline | Sensitivity (95% CI) of Follow–up Responses | Specificity (95% CI) of Follow–up Responses | True Prev (%) Observed at Baseline | Population–Based Survey Estimate at Follow–up | Matched N at Follow–up[b] | AUC at Follow–up (95% CI) | AUC at Baseline (95% CI) | IF at Follow–up | IF at Baseline | Met AUC and IF at Follow–up? [c] |
|---|---|---|---|---|---|---|---|---|---|---|---|
| **Initial Client Assessment** | | | | | | | | | | | |
| tablet) | | | | | | | | | | | |
| Augments labor with a uterotonic (IV, IM, tablet) | 504 | 59.2 (49.1–68.8) | 68.3 (63.5–72.9) | 20.4 | 37.3 | 501 | 0.64* (0.59–0.68) | 0.70 (0.66–0.74) | 1.83 | 1.92 | No |
| Uterotonic received some time before birth (to induce or augment labor) | 499 | 68.1 (58.9–76.3) | 68.4 (63.5–73.1) | 23.8 | 40.3 | 496 | 0.68* (0.64–0.72) | 0.75 (0.71–0.78) | 1.69 | 1.77 | No |
| Performs artificial rupture of the membranes | 507 | 15.6 (11.0–21.2) | 93.9 (90.5–96.3) | 41.8 | 10.1 | 504 | 0.55* (0.50–0.59) | 0.51 (0.47–0.55) | 0.24 | 0.09 | No |

| Indicator | Matched N at Follow–up | Sensitivity (95% CI) of Follow–up Responses | Specificity (95% CI) of Follow–up Responses | True Prev (%) Observed at Baseline | Population–Based Survey Estimate at Follow– | Matched N at Baseline[b] | AUC at Follow–up (95% CI) | AUC at Baseline (95% CI) | IF at Follow up | IF at Baseline | Met AUC and IF at Follow–up? [c] |
|---|---|---|---|---|---|---|---|---|---|---|---|

**Table S2.** Full validation results.[a]

| Indicator | Matched N at Baseline | Sensitivity (95% CI) of Follow–up Responses | Specificity (95% CI) of Follow–up Responses | True Prev (%) Observed at Baseline | Population–Based Survey Estimate at Follow–up | Matched N at Follow–up[b] | AUC at Follow–up (95% CI) | AUC at Baseline (95% CI) | IF at Follow–up | IF at Baseline | Met AUC and IF at Follow–up? [c] |
|---|---|---|---|---|---|---|---|---|---|---|---|
| **Initial Client Assessment** | | | | | | | | | | | |
| | | | | | up | | | | | | |
| **Skilled Birth Attendance** | | | | | | | | | | | |
| Main provider delivery– skilled | 506 | 91.0 (88.0–93.4) | 18.0 (7.5–33.5) | 92.29 | 90.3 | 502 | 0.54 (0.50–0.59) | 0.54 (0.49–0.58) | 0.98 | 1.00 | No |
| Main provider delivery– doctor or med resident | 506 | 77.4 (65.0–87.1) | 76.8 (72.6–80.7) | 12.3 | 29.8 | 502 | 0.77* (0.73–0.81) | 0.86 (0.82–0.89) | 2.44 | 1.57 | No |
| Main provider delivery– nurse/midwife | 506 | 68.6 (63.9–73.1) | 72.3 (62.5–80.7) | 80.0 | 60.5 | 502 | 0.70* (0.66–0.74) | 0.80 (0.76–0.83) | 0.76 | 0.94 | Yes |
| **Second & Third Stage Labor** | | | | | | | | | | | |
| Uterotonic administered in 1–3 minutes following delivery (injection, IV medication, tablets) | 426 | 78.1 (73.3–82.3) | 14.3 (7.6–23.6) | 80.3 | 79.6 | 426 | 0.46 (0.41–0.51) | NA | 1.0 | NA | No |

**Table S2.** Full validation results.[a]

| Indicator | Matched N at Baseline | Sensitivity (95% CI) of Follow–up Responses | Specificity (95% CI) of Follow–up Responses | True Prev (%) Observed at Baseline | Population–Based Survey Estimate at Follow–up | Matched N at Follow–up[b] | AUC at Follow–up (95% CI) | AUC at Baseline (95% CI) | IF at Follow–up | IF at Baseline | Met AUC and IF at Follow–up? [c] |
|---|---|---|---|---|---|---|---|---|---|---|---|
| **Initial Client Assessment** | | | | | | | | | | | |
| (women who had vaginal delivery) | | | | | | | | | | | |
| Uterotonic administered after delivery of placenta (women who had vaginal delivery) | 426 | 54.6 (23.4–83.3) | 64.3 (59.5–68.9) | 2.6 | 36.1 | 426 | 0.59 (0.55–0.64) | 0.49 (0.44–0.54) | 17.24 | 22.09 | No |
| Method of uterotonic post–birth– IM injection (women who had vaginal delivery) | 431 | 75.4 (70.9–79.4) | 53.9 (25.1–80.8) | 97.0 | 74.5 | 438 | 0.65 (0.60–0.69) | NA | 0.77 | NA | Yes |
| Episiotomy | 425 | 75.0 (63.4–84.5) | 84.1 (79.9–87.8) | 16.9 | 25.9 | 434 | 0.80* (0.75–0.83) | 0.86 (0.83–0.89) | 1.53 | 1.21 | No |
| Cesarean section | 509 | 91.0 (81.5–96.6) | 97.7 (95.9–98.9) | 13.2 | 13.9 | 506 | 0.94 (0.92–0.96) | 0.96 (0.94–0.98) | 1.06 | 1.03 | Yes |
| **Immediate Postnatal Care–** | | | | | | | | | | | |

**Table S2.** Full validation results.[a]

| Indicator | Matched N at Baseline | Sensitivity (95% CI) of Follow–up Responses | Specificity (95% CI) of Follow–up Responses | True Prev (%) Observed at Baseline | Population–Based Survey Estimate at Follow–up | Matched N at Follow–up[b] | AUC at Follow–up (95% CI) | AUC at Baseline (95% CI) | IF at Follow–up | IF at Baseline | Met AUC and IF at Follow–up? [c] |
|---|---|---|---|---|---|---|---|---|---|---|---|
| **Initial Client Assessment** | | | | | | | | | | | |
| **Newborn** | | | | | | | | | | | |
| Newborn placed with mother immediately following birth (all women) | 489 | 72.2 (66.6–77.4) | 42.3 (35.5–49.3) | 57.5 | 66.1 | 487 | 0.57 (0.53–0.62) | 0.58 (0.53–0.62) | 1.15 | 1.03 | No |
| Breastfed infant in first hour after birth | 472 | 73.8 (67.8–79.2) | 45.2 (38.6–51.9) | 51.7 | 64.6 | 441 | 0.59 (0.55–0.64) | 0.63 (0.58–0.68) | 1.25 | 1.41 | No |
| Skin to skin (one item) | 489 | 76.2 (60.5–80.8) | 23.6 (22.7–31.5) | 17.0 | 76.4 | 487 | 0.50 (0.45–0.54) | 0.50 (0.45–0.54) | 4.45 | 4.43 | No |
| 3 essential elements of newborn care (immed dried, skin to skin, breastfed) | 472 | 50.0 (33.8–66.2) | 55.3 (50.5–60.1) | 8.5 | 45.1 | 441 | 0.53 (0.48–0.57) | 0.52 (0.47–0.56) | 5.33 | 6.71 | No |

| Indicator | Matched N at Follo | Sensitivity (95% CI) of Follow–up Responses | Specificity (95 CI) of Follow–up Responses | True Prev (%) Observ | Population–Based Survey | Matched N at Baseli | AUC at Follow–up (95% CI) | AUC at Baseline (95% CI) | IF at Follow up | IF at Baseline | Met AUC and IF at |
|---|---|---|---|---|---|---|---|---|---|---|---|

**Table S2.** Full validation results.[a]

| Indicator | Matched N at Baseline | Sensitivity (95% CI) of Follow–up Responses | Specificity (95% CI) of Follow–up Responses | True Prev (%) Observed at Baseline | Population–Based Survey Estimate at Follow–up | Matched N at Follow–up[b] | AUC at Follow–up (95% CI) | AUC at Baseline (95% CI) | IF at Follow–up | IF at Baseline | Met AUC and IF at Follow–up? [c] |
|---|---|---|---|---|---|---|---|---|---|---|---|
| **Initial Client Assessment** | | | | | | | | | | | |
| | w–up | | | ed at Baseline | Estimate at Follow–up | ne[b] | | | | | Follow–up? [c] |
| **Immediate Postnatal Care–Mother** | | | | | | | | | | | |
| Uterine massage after delivery of placenta (denominator: vaginal delivery) | 429 | 68.5 (62.8–73.7) | 29.0 (21.4–37.6) | 69.5 | 69.2 | 436 | 0.49 (0.44–0.54) | 0.51 (0.46–0.56) | 1.00 | 1.26 | No |
| In first examination post–delivery, did provider ask or check for bleeding? | 500 | 56.9 (52.2–61.5) | 42.2 (27.7–57.8) | 57.0 | 57.3 | 486 | 0.50* (0.45–0.54) | 0.38 (0.34–0.43) | 1.01 | 0.67 | No |
| In first examination post–delivery, did provider examine | 500 | 26.8 (22.7–31.1) | 76.3 (63.4–86.4) | 88.2 | 26.4 | 476 | 0.52 (0.47–0.56) | 0.57 (0.53–0.62) | 0.30 | 0.57 | No |

**Table S2.** Full validation results.[a]

| Indicator | Matched N at Baseline | Sensitivity (95% CI) of Follow–up Responses | Specificity (95% CI) of Follow–up Responses | True Prev (%) Observed at Baseline | Population–Based Survey Estimate at Follow–up | Matched N at Follow–up[b] | AUC at Follow–up (95% CI) | AUC at Baseline (95% CI) | IF at Follow–up | IF at Baseline | Met AUC and IF at Follow–up? [c] |
|---|---|---|---|---|---|---|---|---|---|---|---|
| **Initial Client Assessment** | | | | | | | | | | | |
| perineum? | | | | | | | | | | | |
| In first examination post–delivery, did provider take temperature? | 504 | 46.3 (39.4–53.4) | 56.2 (50.4–61.9) | 40.7 | 44.8 | 502 | 0.51* (0.47–0.56) | 0.62 (0.57–0.66) | 1.10 | 1.48 | No |
| In first examination post–delivery, did provider take blood pressure? | 504 | 50.2 (43.7–56.7) | 55.9 (49.7–62.1) | 48.2 | 47.0 | 502 | 0.53* (0.49–0.57) | 0.62 (0.57–0.66) | 0.98 | 1.58 | No |
| In first examination post–delivery, did provider check for involution? | 490 | 40.1 (35.1–45.2) | 57.4 (47.5–66.9) | 78.0 | 40.6 | 479 | 0.49 (0.44–0.53) | 0.52 (0.47–0.56) | 0.52 | 0.80 | No |
| **Maternal and Newborn Morbidity** | | | | | | | | | | | |
| Low birthweight infant (<2500g) | 490 | 68.1 (52.9–80.9) | 95.0 (92.6–96.9) | 9.59 | 11.0 | 463 | 0.82 (0.78–0.85) | 0.87 (0.83–0.90) | 1.15 | 0.93 | Yes |

**Table S2.** Full validation results.[a]

| Indicator | Matched N at Baseline | Sensitivity (95% CI) of Follow–up Responses | Specificity (95% CI) of Follow–up Responses | True Prev (%) Observed at Baseline | Population–Based Survey Estimate at Follow–up | Matched N at Follow–up[b] | AUC at Follow–up (95% CI) | AUC at Baseline (95% CI) | IF at Follow–up | IF at Baseline | Met AUC and IF at Follow–up? [c] |
|---|---|---|---|---|---|---|---|---|---|---|---|
| **Initial Client Assessment** | | | | | | | | | | | |
| Complications–hemorrhage | 508 | 50.0 (28.2–71.8) | 83.3 (79.7–86.5) | 4.3 | 18.1 | 505 | 0.67 (0.62–0.71) | 0.63 (0.59–0.67) | 4.18 | 2.64 | No |
| Complications–prolonged labor | 508 | 65.0 (40.8–84.6) | 75.0 (70.9–78.8) | 3.9 | 26.6 | 505 | 0.70 (0.66–0.74) | 0.61 (0.56–0.65) | 6.75 | 6.15 | No |
| Complications– none | 508 | 51.1 (46.4–55.8) | 71.4 (57.8–82.7) | 89.0 | 48.6 | 505 | 0.61 (0.57–0.65) | 0.58 (0.53–0.62) | 0.55 | 0.57 | No |
| Complications– yes (to any) | 508 | 71.4 (57.8–82.7) | 52.4 (47.7–57.1) | 11.0 | 50.2 | 505 | 0.62 (0.58–0.66) | 0.59 (0.55–0.63) | 4.55 | 4.04 | No |
| Asked for pain relief medication | 505 | 47.0 (32.1–61.9) | 64.0 (59.4–68.4) | 9 | 37.0 | 498 | 0.55 (0.51–0.60) | 0.54 (0.49–0.58) | 3.98 | 3.55 | No |
| Received pain relief medication | 506 | 82.0 (72.5–89.4) | 44.4 (39.5–49.3) | 18 | 60.3 | 499 | 0.63 (0.59–0.67) | 0.67 (0.63–0.71) | 3.43 | 3.30 | No |

[a] Validation findings for all indicators with sufficient cell counts at follow–up.

[b] To allow for observed changes among the same women over time, baseline validation results restricted to women who participated in follow–up study.

[c] Indicators had moderate or high accuracy AUC ( >0.60) and low bias IF (0.75<IF<1.25).

[*] Statistically different AUC values between baseline and follow–up at p<0.05.

Notes: NA results had insufficient data (n<5 in cell of two by two table) for robust analysis.

Table S3. Reliability of women's reports for indicators[a].

| Indicator | N | Test–rest reliability, $r_{phi}$ |
|---|---|---|
| **Initial Client Assessment** | | |
| Takes blood pressure– initial examination | 512 | 0.15 |
| HIV status checked | 512 | 0.14 |
| Receives HIV test | 508 | 0.14 |
| Providers washes hands with soap and water or uses antiseptic before any initial examination | 512 | 0.08 |
| **Provider Respectful Care** | | |
| Encourages/assists woman to ambulate during labor | 512 | 0.05 |
| Allowed to drink liquids/ eat | 512 | 0.25 |
| Encourages/assists woman to assume different positions in labor | 512 | 0.12 |
| Allowed to have a support person present during labor and delivery | 512 | 0.17 |
| A support person/companion is present during birth | 512 | 0.47 |
| **First Stage of Labor** | | |
| Induces labor with a uterotonic (IV, IM, tablet) | 512 | 0.33 |
| Augments labor with a uterotonic (IV, IM, tablet) | 512 | 0.44 |
| Uterotonic received some time before birth (to induce or augment labor) | 512 | 0.50 |
| Performs artificial rupture of the membranes | 512 | 0.15 |
| **Skilled Birth Attendance** | | |
| Main provider delivery– skilled | 511 | 0.03 |
| Main provider delivery– doctor or medical resident | 511 | 0.32 |
| Main provider delivery– nurse/midwife | 511 | 0.32 |

| Indicator | N | Test–rest reliability, $r_{phi}$ |
|---|---|---|
| **Second & Third Stage Labor** | | |
| Uterotonic administered in 1–3 minutes following delivery (injection, IV medication, tablets) (women who had vaginal delivery) | 437 | 0.02 |
| Uterotonic administered after delivery of placenta (women who had vaginal delivery) | 437 | 0.08 |
| Method of uterotonic post–birth– IM injection (women who had vaginal delivery) | 432 | 0.11 |
| Episiotomy | 433 | 0.63 |
| Cesarean section | 512 | 0.90 |
| **Immediate Postnatal Care– Newborn** | | |
| Newborn placed with mother immediately following birth (all women) | 509 | 0.21 |
| Breastfed infant in first hour after birth | 446 | 0.33 |
| Skin to skin newborn care | 510 | 0.53 |
| 3 essential elements of newborn care (immed dried, skin to skin, breastfed) | 446 | 0.22 |
| **Immediate Postnatal Care– Mother** | | |
| Uterine massage after delivery of placenta (women who had vaginal delivery) | 432 | 0.09 |
| In first examination post–delivery, did provider ask or check for bleeding? | 493 | 0.15 |
| In first examination post–delivery, did provider examine perineum? | 485 | 0.04 |
| In first examination post–delivery, did provider take temperature? | 509 | 0.16 |

| Indicator | N | Test–rest reliability, $r_{phi}$ |
|---|---|---|
| In first examination post–delivery, did provider take blood pressure? | 509 | 0.14 |
| In first examination post–delivery, did provider check for involution? | 475 | 0.03 |
| **Maternal and Newborn Morbidity** | | |
| Low birthweight infant (<2500g) | 458 | 0.71 |
| Complications– hemorrhage | 512 | 0.20 |
| Complications– prolonged labor | 512 | 0.21 |
| Complications– none | 512 | 0.16 |
| Complications– yes (to any) | 512 | 0.17 |
| Asked for pain relief medication | 506 | 0.23 |
| Received pain relief medication | 506 | 0.38 |

[a] Analysis performed for indicators for which there were at least 5 counts per cell of two–by–two table comparing women's self–reports at follow–up and baseline.
